# *Neisseria meningitidis* IgA1-specific serine protease exhibits novel cleavage activity against IgG3

**DOI:** 10.1080/21505594.2021.1871822

**Published:** 2021-01-18

**Authors:** Christian Spoerry, Jens Karlsson, Marie-Stephanie Aschtgen, Edmund Loh

**Affiliations:** a Department of Microbiology, Tumor, and Cell Biology, BioClinicum, Karolinska University Hospital, Stockholm, Sweden; b Singapore Centre for Environmental Life Sciences Engineering, Nanyang Technological University, Singapore, Singapore

**Keywords:** Meningococccus, IgA, IgG3, protease, IgA1-specific serine protease, *Neisseria meningitidis*, immunoglobulin, immune evasion, IMD, invasive meningococcal disease

## Abstract

*Neisseria meningitidis* (meningococcus) is a common bacterial colonizer of the human nasopharynx but can occasionally cause very severe systemic infections with rapid onset. Meningococci are able to degrade IgA encountered during colonization of mucosal membranes using their IgA1-specific serine protease. During systemic infection, specific IgG can induce complement-mediated lysis of the bacterium. However, meningococcal immune evasion mechanisms in thwarting IgG remain undescribed. In this study, we report for the first time that the meningococcal IgA1-specific serine protease is able to degrade IgG3 in addition to IgA. The IgG3 heavy chain is specifically cleaved in the lower hinge region thereby separating the antigen binding part from its effector binding part. Through molecular characterization, we demonstrate that meningococcal IgA1-specific serine protease of cleavage type 1 degrades both IgG3 and IgA, whereas cleavage type 2 only degrades IgA. Epidemiological analysis of 7581 clinical meningococcal isolates shows a significant higher proportion of cleavage type 1 among isolates from invasive cases compared to carrier cases, regardless of serogroup. Notably, serogroup W cc11 which is an increasing cause of invasive meningococcal disease globally harbors almost exclusively cleavage type 1 protease. Our study also shows an increasing prevalence of meningococcal isolates encoding IgA1P cleavage type 1 compared to cleavage type 2 during the observed decade (2010–2019). Altogether, our work describes a novel mechanism of IgG3 degradation by meningococci and its association to invasive meningococcal disease.

## Introduction

*Neisseria meningitidis* is a common bacterial transient colonizer of the human nasopharynx. By mechanisms not fully understood, harmless colonization can rapidly turn into an invasive infection leading to lethal septicemia and meningitis [1]. Despite antimicrobial treatment, invasive meningococcal disease (IMD) is still fatal in up to 15% of all cases, with high morbidity among the survivors [2,3]. The meningococcus utilizes various virulence factors to facilitate its survival in the host. These include the polysaccharide capsule that enables the pathogen to evade complement-mediated killing and the Type 4 pili (T4P) for adhesion to mucosal membranes of the nasopharynx during colonization as well as microcolony formation [4].

The host mucosal membranes where meningococci colonize are immersed in secretory IgA that can limit or clear infectious agents as a first line of defense. Specific IgA antibodies can neutralize and agglutinate pathogens, as well as induce opsonophagocytosis and polymorphonuclear neutrophil (PMN) respiratory burst [5,6]. In 1975, it was first described that a meningococcal extracellular enzyme later termed IgA1-specific serine protease (IgA1P) is capable of specifically degrading human IgA1 [7]. As a member of the autotransporter protein family, IgA1Ps maturation involves several autoproteolytic cleavage steps leading to secretion of the approximately 100 kDa protease domain [8,9]. The meningococcal IgA1P cleaves IgA1 in the hinge region either between a proline and a serine (referred to as IgA1P cleavage type 1) or two aa closer to the N-terminal between a proline and a threonine (referred to as IgA1P cleavage type 2) [10]. A genome comparative study of the meningococcal *iga* gene encoding for IgA1P revealed that cleavage type 1 and cleavage type 2 variants are defined by two conserved, but distinctly different sequences in the protease domain between aa 58 and 344 [11]. In addition to cleaving IgA1, the meningococcal IgA1P has also been reported to degrade lysosome-associated membrane protein 1 (Lamp1) [12] and transcription factor NFκB p65 [13]. The pleotropic immune modulating functions of IgA1P suggest that it might play a pivotal role during meningococcal infection.

The convergent evolution of IgA degrading proteases among other host-specific pathogens such as *Streptococcus pneumoniae* and *Haemophilus influenzae* residing in similar niches further strengthens the biological importance of IgA1 degrading proteases [14]. IgA1 cleavage can potentially inhibit agglutination, opsonophagocytosis, and PMN respiratory burst, as these immune response mechanisms are mediated by IgA [5,6]. Additionally, generated IgA Fab fragments can cover antigens on the bacterial surface, shielding them from opsonising antibodies of other immunoglobulin classes (*i.e*. fabulation) [15].

However, IgA1 cleavage does not affect initiation of the classical complement pathway, as IgA1 lacks a C1q-binding motif, in contrast to IgG and IgM. Specific antibodies of the IgG1 and IgG3 subclasses are especially effective in initiating complement-mediated killing of meningococci [5]. To evade antibody-mediated immunity, many host-specific pathogens secrete highly specific IgG or IgM degrading proteases [16–19]. To date, there is no other immunoglobulin degrading protease reported in the meningococcus besides the IgA1 degrading IgA1P. As the meningococcus encounters other immunoglobulins during systemic infection, we investigated whether the meningococcus could also degrade IgG or IgM antibodies. Here we report for the first time that meningococci are able to degrade IgG3 antibodies mediated by its IgA1P cleavage type 1. To study if IgG3 degradation could contribute to immune evasion, the association between IMD cases and IgA1P cleavage type 1 encoding isolates was investigated.

## Materials and methods

### Bacterial strains and growth conditions

*N. meningitidis* was grown in Brain Heart Infusion broth (BHI, Sigma-Aldrich, 37 g dissolved in 1 L dH_2_O) or on BHI agar (Sigma-Aldrich) (1.5% w/v) supplemented with 1 g/L starch (Sigma-Aldrich) and 5% heat-inactivated horse blood lysate (Thermo Scientific). Bacteria on solid support were grown at 37°C with 5% CO_2_. When appropriate, BHI agar was supplemented with 2.5 µg/mL erythromycin. Liquid cultures were inoculated to an initial OD_600_ of ~0.05 and grown at 37°C under agitation (130 rpm). *E. coli* was grown on Lysogeny Agar (LA) when appropriate supplemented with 50 µg/mL kanamycin at 37°C. Source and characteristics of bacterial strains used in this study can be found in Table 1.

**Table 1.** Bacterial strains used in this study

**Strain**	**Comments**	**IgA1P cleavage type**	**Reference**
*N. meningitidis* MC58	Serogroup B, ATCC BAA-335, ST-32 complex	1	[50]
*N. meningitidis* 18–174	id: 61,298, Serogroup B, ST-32 complex	2	clinical isolates provided by Susanne Jacobsson, Department of Laboratory Medicine, Clinical Microbiology, Örebro University Hospital, Sweden
*N. meningitidis* 16–102	id: 41,968, Serogroup B, ST-41/44 complex	2	
*N. meningitidis* 15–25	id: 42,410, Serogroup B, ST-213 complex	2	
*N. meningitidis* H44/76	Serogroup B, ST-32 complex	1	[51]
*N. meningitidis* FAM18	Serogroup C, ATCC 700,532, ST-11 complex	1	[52]
*N. meningitidis* Z2491	Serogroup A, ST-4 complex	1	[53]
*N. meningitidis* MC58 Δ*iga*	replacement of NMB0700 with erythromycin resistance-cassette	-	this study
*E. coli* BL21(DE3) Rosetta T1^R^	F *ompT hsdS_B_*(r_B_^−^ m_B_^−^) *gal dcm* ≠ (DE3) *tonA*	-	purchased from Sigma-Aldrich

### DNA techniques and primer sequences

Primers were designed based on the genomes of *N. meningitidis* MC58, FAM18 and isolate 18–174. All PCRs were conducted with Phusion Master Mix HF (Thermo Scientific). Obtained strains and plasmids were confirmed by PCR, gel electrophoresis and sequencing. Primers and plasmids used for this study can be found in Table 2.

**Table 2.** Oligonucleotides and plasmids used in this study

Oligonucleotide	Description	Sequence (5´-3´)^a^
**PCR primers to generate MC58 Δ*****iga***		
iga-5 f	to amplify 5´ region of NMB0700 in MC58	*ATGCCGTCTGAAGAT*CACTATAAGAATGCAAACGAAAAGCC
iga-5 r		GGTGCAAGTCACACGAACACGAAGATCATTCATTTTGGTGTTTTATTGCC
iga-erm-f	to amplify erythromycin-resistance cassette	GGCAATAAAACACCAAAATGAATGATCTTCGTGTTCGTGTGACTTGCACC
iga-erm-r		GGCATTTTATTTTGCTATGAATTTAGGGATGTTGCTGATTAAGACGAGC
iga-3 f	to amplify 3´ region of NMB0700 in MC58	GCTCGTCTTAATCAGCAACATCCCTAAATTCATAGCAAAATAAAATGCC
iga-3 r		*ATGCCGTCTGAACT*GCACGCTTCGGTATGACTTGC
**PCR primers to generate FAM18 Δ*iga***		
iga-5 f	to amplify 5´ region of NMC0651 in FAM18	*ATGCCGTCTGAAGAT*CACTATAAGAATGCAAACGAAAAGCC
FAM18iga_5erm_r2		GAGAATATTTTATATTTTTGTTCATAAGGTTTTACCGTTTTAAGGGTG
FAM18iga_erm_f2	to amplify erythromycin-resistance cassette	CACCCTTAAAACGGTAAAACCTTATGAACAAAAATATAAAATATTCTC
FAM18iga_839erm_r3		GTTTGCGTGCGTTTCGAACTAAACCTTATTTCCTCCCGTTAAATAATAG
FAMM18iga_839erm_f3	to amplify 3´ region of NMC0651 in FAM18	CTATTATTTAACGGGAGGAAATAAGGTTTAGTTCGAAACGCACGCAAAC
FAM18iga_839_r2		*ATGCCGTCTGAACT*GTGGACGACACGTTCTACATCATCG
**PCR primers to clone protease domain encoding sequence of the *iga* genes into pNIC-CTHF plasmids**		
FWD-11,253	to amplify *iga* nt 81–3021 from MC58	TTAAGAAGGAGATATACTATGGCGGCATTGGTCAGAGACG
REV-11,202		GATTGGAAGTAGAGGTTCTCTGCAGGCGGTGCGACGACGAT
iga1p-vec-f	to amplify plasmid backbone from pNIC-CTHF-rIgA1P-ct1	GCAGATGAAATCAAACAACG
iga1p-vec-r		CAACGCTAAAATCAATCATCG
iga1p-ins-f	to amplify *iga* nt 222–977 from 18–174	GATGATTGATTTTAGCGTTGC
iga1p-ins-r		CGTTGTTTGATTTCATCTGC
**Plasmid**	**Description**	**Reference**
pSp72/*LytA-Erm*	Plasmid template to obtain the erythromycin resistance cassette.	[54]
pNIC-CTHF	empty vector used for cloning of plasmids below	[55]
pNIC-CTHF-rIgA1P-ct1	for production of recombinant IgA1P cleavage type 1 (from strain MC58)	this study
pNIC-CTHF-rIgA1P-ct2	for production of recombinant IgA1P cleavage type 2 (from strain 18–174)	this study

^a^The *Neisseria* uptake sequence is in *italic*.

### Generation of *N. meningitidis* ∆iga deletion mutant strains

Deletion mutant strains MC58 ∆*iga* (NMB0700) and FAM18 ∆*iga* (NMC0651) were generated as described in Righetti *et al*. [20]. Briefly, a ~ 1 kb 5´ amplicon of *iga*, an amplicon of an erythromycin resistance cassette and a ~ 1 kb 3´ amplicon were assembled by the Gibson Assembly system (New England Biolabs). The assembled fragments were amplified by PCR with the flanking primers containing the neisserial DNA uptake sequence [21] and then introduced into *N. meningitidis* MC58 or FAM18 by natural transformation. Through homologous recombination, the gene of interest was replaced by the erythromycin resistance cassette. Deletion mutant clones were selected on BHI agar containing erythromycin and confirmed by selective PCR analysis and sequencing of genomic amplicons.

### Sequence analysis to identify IgA1P cleavage type 1 and cleavage type 2 encoding strains

Meningococcal strains were classified as either as IgA1P cleavage type 1 or cleavage type 2 encoding strains. The classification was based on the presence or absence of the aa consensus sequence motif VYEKGAYHQEGNEKG in translated sequences of the *iga* gene locus NEIS0651 on the *Neisseria* MultiLocus Sequence Typing website (https://pubmlst.org/neisseria/) sited at the University of Oxford [22] or of *iga* gens deposited on NCBI (https://www.ncbi.nlm.nih.gov/genome). The motifs are in accordance with aligned aa sequences of IgA1P published by Lomholt *et al*. [11] and present exclusively in the protease domain of IgA1P cleavage type 2.

Strains already present in our laboratory were identified as IgA1P cleavage type 1 encoding strains. Clinical isolates from Sweden encoding IgA1P cleavage type 2 were identified on the *Neisseria* MultiLocus Sequence Typing website and kindly supplied by Dr. Susanne Jacobsson, Department of Laboratory Medicine, Clinical Microbiology, Örebro University Hospital, Sweden (Table S1). Sequence alignments were performed in CLC Main Workbench Version 8.0.1 software (Qiagen). The protease domains of IgA1P cleavage type 1 and 2 were identified using the Pfam database [23]. These domains were then *in silico* modeled by SWISS-MODEL [24] using a crystal structure of *Haemophilus influenzae* IgA1 protease [25] as a template and the aa sequences of IgA1P cleavage type 1 and 2 deposited in the supplemental data.

### Cloning, production and purification of recombinant IgA1P cleavage type 1 and cleavage type 2

The protease domain encoding sequence of the *iga* gene (aa 27 to 1007) was amplified from chromosomal DNA of *N. meningitidis* MC58 (for cleavage type 1) using primers FWD-11,253 and REV-11,202 and cloned into pNIC-CTHF by ligation-independent cloning [26] resulting in a plasmid encoding C-terminally 6xHis tagged IgA1P protease domain designated pNIC-CTHF-rIgA1P-ct1. To construct pNIC-CTHF-rIgA1P-ct2, parts of the *iga* gene from strain 18–174 were amplified using primers iga1p-ins-f and iga1p-ins-r, and cloned into pNIC-CTHF-rIgA1P-ct1 by FastCloning [27] using primers iga1p-vec-f and iga1p-vec-r to amplify the vector backbone. The sequence of the *iga* gene originating from MC58 in pNIC-CTHF-rIgA1P-ct2 was identical to the sequence of 18–174. Entire inserts were sequenced to verify the cloning and the sequences of *iga*.

*E. coli* BL21(DE3) Rosetta T1R (Invitrogen) carrying the plasmid pNIC-CTHF-rIgA1P-ct1 and pNIC-CTHF-rIgA1P-ct2 were grown in Terrific Broth with 8 g/L glycerol in a LEX bioreactor (Harbinger Biotech) at 37°C until OD_600_ of ~2. Temperature was then reduced to 18°C prior to induction of protein production with 0.5 mM IPTG for 16 hours. Cells were harvested and cell pellet was resuspended in Lysis buffer (100 mM HEPES, 500 mM NaCl, 10 mM Imidazole, 10% glycerol, 0.5 mM TCEP, pH 8.0 and 250 U/µl Benzonase, Merck) and sonicated (Sonics Vibra-cell) at 70% amplitude, 3 s on/off for 3 min, on ice. The lysate was clarified by centrifugation and filtration. Purification was conducted on an ÄKTAXpress system (GE Healthcare). The lysates were loaded on Ni-NTA Superflow (Qiagen), washed with wash buffer 1 (20 mM HEPES, 500 mM NaCl, 10 mM Imidazole, 10% (v/v) glycerol, 0.5 mM TCEP, pH 7.5) and wash buffer 2 (20 mM HEPES, 500 mM NaCl, 25 mM Imidazole, 10% (v/v) glycerol, 0.5 mM TCEP, pH 7.5) until a stable baseline was obtained. Proteins were eluted with elution buffer (20 mM HEPES, 500 mM NaCl, 500 mM Imidazole, 10% (v/v) glycerol, 0.5 mM TCEP, pH 7.5) and stored in sample loops on the system and then injected into HiLoad 16/60 Superdex 200 prep grade (GE Healthcare) for gel filtration using a buffer containing 20 mM HEPES, 300 mM NaCl, 10% (v/v) glycerol, 0.5 mM TCEP, pH 7.5. Elution peaks were collected in 2 mL fractions and analyzed on SDS-PAGE gels. The entire purification was performed at 4°C. Protein samples were concentrated by Vivaspin 20 filter concentrators (30 K MWCO, VivaScience) at 15°C to 11.71 mg/mL (cleavage type 1) and 6.99 mg/mL (cleavage type 2) prior to storage at −80°C. The final protein concentration was assessed by Nanodrop ND-1000 (Nano-Drop Technologies).

### Immunoglobulin degradation assay

To screen for immunoglobulin cleavage by *N. meningitidis*, bacteria were cultured in presence of 2% heat-inactivated human serum (containing immunoglobulins) to stationary phase. Cultures were spun down and supernatants were used to prepare samples for SDS-PAGE and subsequent western blot analysis.

To investigate cleavage specificity toward human immunoglobulins, sterile filtered supernatants of stationary phase cultures or 0.02 to 0.1 mg/mL rIgA1P in PBS were incubated with 0.25 mg/mL IgA (purified from serum; Sigma-Aldrich), IgG1 kappa, IgG2 kappa, IgG3 kappa and IgG4 kappa (all purified from myeloma serum; Sigma-Aldrich) at 37°C for 16 hours, if not otherwise stated. Samples were then subjected to SDS-PAGE. Due to discontinuation of the product, IgG4 was substituted with Nivolumab (Bristol-Myers Squibb) in experiments with rIgA1P.

### SDS-PAGE and Western blot analyses

Samples for SDS-PAGE were prepared with either reducing (containing 2-Mercaptoethanol, Sigma-Aldrich) or non-reducing SDS-PAGE sample buffer and heated to 95°C for 5 minutes prior to electrophoresis on NuPAGE 4–12% Bis-Tris protein gels (Invitrogen) or Criterion TGX Precast Gels (Bio-Rad). Novex Sharp pre-stained protein standard (Thermo Scientific) was used as protein size ruler. Gels were either stained by Imperial protein stain (Thermo Scientific) or blotted to Hybond-P PVDF membranes (GE Healthcare), using a Trans-Blot Turbo system (Bio-Rad) for western blot analysis.

Membranes were blocked for 1 h with 5% dry milk powder in PBS with 0.1% Tween (PBST), followed by incubation with horseradish peroxidase-conjugated antibodies for 1 h. Goat anti-Human IgA-HRP (A18781, Thermo Scientific) and goat anti-Human IgM-HRP (A18835, Invitrogen) were diluted 1/10,000 and Goat anti-Human IgG Fc-HRP (A18817, Invitrogen) was diluted 1/20,000. Membranes were thoroughly washed with PBST prior to development with Amersham Biosciences ECL Select Western blotting detection reagent (GE Healthcare) according to manufacturer’s instruction and visualized by GelDoc XRS+ (Bio-Rad).

### Identification of cleavage site by N-terminal Edman sequencing

IgG3 (final concentration 0.25 mg/mL) was processed through incubation with *N. meningitidis* MC58 stationary phase culture supernatant for 16 h at 37°C, separated by SDS-PAGE under reducing conditions as previously explained and transferred by semi-dry blotting to Hybond-P PVDF membrane (GE Healthcare) with 50 mM sodium borate, 20% methanol as blotting buffer at 4°C. The membrane was stained with coomassie (0.1% CBB R250 (Sigma), 10% acetic acid, 40% methanol in Milli-Q water). After destaining, the degradation products were tightly excised and subjected to N-terminal Edman sequencing performed by Proteome Factory (Berlin, Germany). The aa sequence APELLGGP was obtained for the smaller (∼32 kDa) degradation product corresponding to the lower hinge region of the human IgG3 heavy chain.

### Epidemiological comparison of IgA1P cleavage type 1 and cleavage type 2 encoding clinical isolates between IMD and carrier cases

*N. meningitidis* clinical isolates collected between 2010 and 2019 in Europe and deposited in the *Neisseria* MultiLocus Sequence Typing website were identified using parameters “Continent” (=Europe) and “Year” (≥2010, ≤2019) in the search query. Translated aa sequences of the *iga* gene locus NEIS0651 and additionally deposited information of the identified isolates were downloaded (April 16 2020) and imported to Microsoft Excel. Isolates with complete aa sequences (n = 8203) were classified as either IgA1P cleavage type 1 or cleavage type 2 encoding isolates based on the same sequence requirement as mentioned above. Isolates with no sequence for NEIS0651 (n = 3280) or early stop codons (n = 2) were excluded from subsequent analysis. Isolates were either grouped as isolates from carrier cases (n = 624) if “disease” was “carrier” or as isolates from IMD cases (n = 6957) if “disease” was “septicaemia,” “meningitis,” “invasive__unspecified/other” or “meningitis_and_septicaemia” in this database. All isolates fulfilling the investigated criteria were analyzed for frequency and prevalence in contingency tables. This analysis was also stratified for isolates of the most common serogroups (B, C, Y, W and non-groupable) as well as most common clonal complexes (cc11, cc31/44, cc23, cc269, cc32 and cc213). Statistical analysis was performed as mentioned below.

Genomic relationship analysis was performed using MLST profiles extracted from PubMLST for isolates from either carrier or IMD cases. Visualization was done using GrapeTree with unrooted minimum spanning tree relationships calculated through MSTree V2 [28]. All branches with nodes below a distance of 2 were collapsed for clear visualization. Metadata for cleavage type 1 and 2 frequencies was imported and visualized as an overlay of the original phylogenetic tree.

### Statistics

To assess significance differences between the prevalence of IgA1P cleavage type 1 or cleavage type 2 encoding isolates between carrier and IMD cases, Fisher’s exact test was performed. Correlation between prevalence of IgA1P cleavage type 1 encoding isolates and time (year of investigation) was assessed by Pearson correlation. All statistical analyses were conducted using GraphPad Prism Version 7.0. A *p*-value <0.05 was considered as significant. Numbers underlying these analysis and exact *p*-values are reported in Table S1.

## Results

### Meningococcal IgA1P mediates cleavage of IgG heavy chain

**Figure 1.**
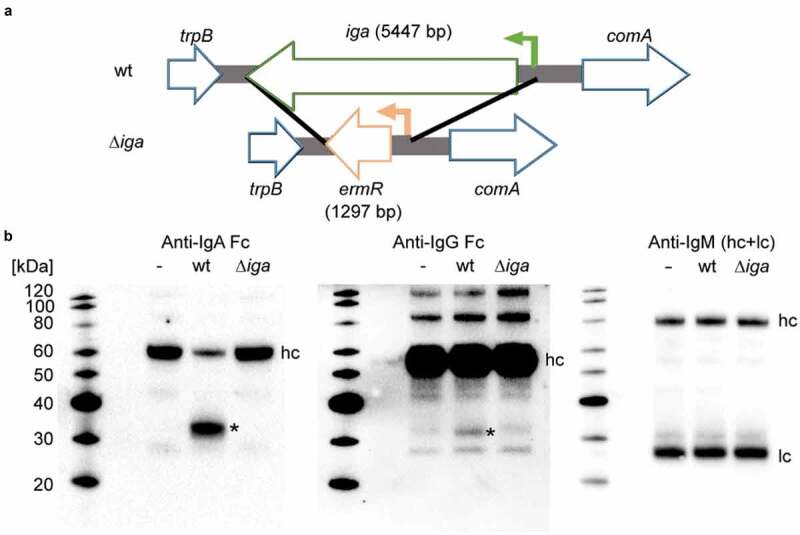
Degradation of immunoglobulins in human serum by *N.* *meningitidis*. (a) An isogenic *iga* deletion mutant strain (*∆iga*) was constructed through allelic replacement of the *iga* gene with an erythromycin resistance cassette. (Green arrow denotes *iga* promoter and orange arrow denotes *ermR* promoter) (b) Western blot analysis under reducing conditions of 2% heat-inactivated human serum alone (-), incubated with *N. meningitidis* MC58 (wt) or its isogenic *∆iga* mutant during growth to stationary phase. Degradation of immunoglobulins was detected with immunoglobulin isotype specific HRP conjugated antibodies. Observed IgA and IgG degradation products are indicated with asterisks (*) as well as heavy chains (hc) and light chains (lc). Each western blot analysis was repeated independently three times and representative blots are displayed

To examine if the meningococcus possesses other immunoglobulin degrading capacities than the well described IgA1 degradation by IgA1P [7], an isogenic IgA1P knock-out mutant strain (*∆iga*) was generated in the *N. meningitidis* MC58 wild type (wt) background trough allelic replacement of the *iga* gene with an erythromycin resistance cassette (Figure 1(a)). Both the parental MC58 wt and MC58 *∆iga* mutant strains had similar growth rates (Fig. S1). Next, these two strains were cultured in presence of 2% heat inactivated pooled human serum to stationary phase. Thereafter, the growth culture supernatants were harvested and subjected to western blot analysis with specific antibodies against major human immunoglobulins (IgA, IgG and IgM) (Figure 1(b)). When MC58 wt was cultured with human serum, degradation of the IgA heavy chain and the appearance of a ~ 32 kDa product were observed. This degradation product did not appear when MC58 *∆iga* was cultured with human serum. In addition to this expected IgA degradation, we observed the appearance of a minor IgG degradation product of ~29 kDa when MC58 wt was cultured with human serum. This observed degradation product suggests cleavage of the IgG heavy chain as the anti-IgG antibody used is specific for the Fc region of the IgG heavy chain. This degradation product did not appear when MC58 ∆*iga* was cultured with human serum suggesting that IgA1P mediates directly or indirectly in the IgG degradation. Degradation of IgM was neither observed when MC58 wt nor ∆*iga* were cultured with human serum.

### IgG3 is cleaved between the core and lower hinge region

**Figure 2.**
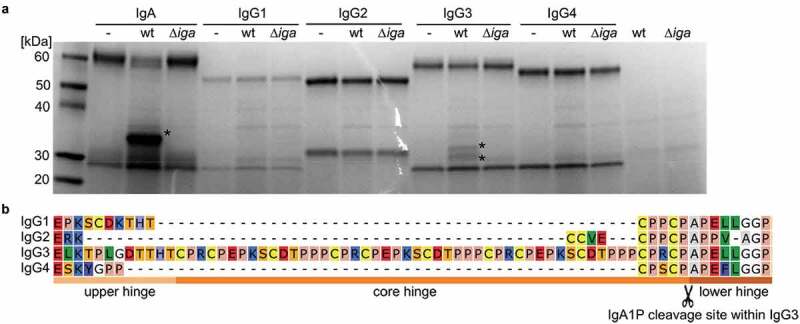
IgA1P-mediated immunoglobulin cleavage specificity and cleavage site within IgG3. (a) Reducing SDS-PAGE analysis of IgA and IgG1 to 4 incubated either with stationary phase culture supernatants of *N. meningitidis* MC58 (wt) or its isogenic ∆*iga* mutant. Observed IgA and IgG3 degradation products are indicated with asterisks (*). (b) aa sequence alignment of the hinge regions of IgG subclasses. The IgA1P cleavage site between the core and lower hinge region of IgG3 identified by N-terminal sequencing of the ~ 29 kDa degradation product is indicated (scissor symbol)

As only a minor fraction of the human IgG was degraded, we hypothesize that the observed IgG degradation mediated by IgA1P might be IgG subclass specific. To investigate this, purified human IgG subclasses 1 to 4 and IgA were incubated with sterile filtered supernatants of meningococcal wt and ∆*iga* stationary phase cultures and then analyzed by SDS-PAGE. Besides degradation of IgA, IgG3 degradation was observed when purified immunoglobulins were incubated with culture supernatant of MC58 wt (Figure 2(a)). In addition to the ~29 kDa degradation product previously detected by anti-IgG Fc western blot analysis (Figure 1), a higher molecular weight IgG3 degradation product of ~31 kDa was observed.

To determine the cleavage site within the IgG3 heavy chain, these two degradation products were excised and analyzed by N-terminal Edman sequencing. No sequence was detected for the ~31 kDa degradation product indicating that its N-terminal was blocked, as expected for the native N-terminal of the IgG3 heavy chain. For the ~29 kDa degradation product, the N-terminal aa sequence APELLGGP corresponding to the lower hinge region of the IgG3 heavy chain was identified (Figure 2(b)). Sequence alignments of the hinge regions of all four human IgG subclasses reveal that all subclasses contain similar aa sequences close to the cleavage site, but only IgG3 is preceded by an elongated core hinge (Figure 2(b)).

### IgG3 is cleaved in a sequential manner into F(ab)2 and ½ Fc fragments

**Figure 3.**
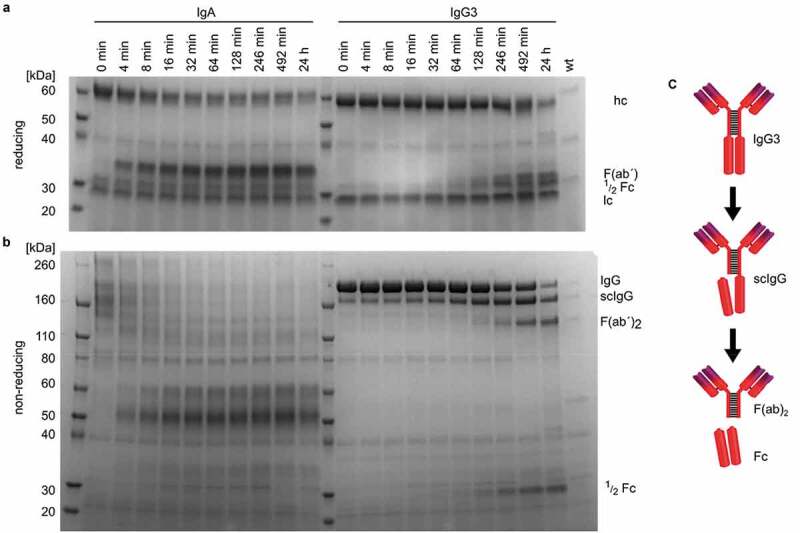
Time course cleavage of IgA and IgG3 by meningococcal culture supernatant and IgG3 cleavage model. (a) Reducing and (b) non-reducing SDS-PAGE analysis of IgA and IgG3 incubated with stationary phase culture supernatants of *N. meningitidis* MC58 (wt) at 37°C. Samples were taken from the reaction at indicated time points. The sizes of IgG3 heavy chain (hc), light chain (lc) and the IgG3 degradation products and intermediates single cleaved IgG3 (scIgG3), F(ab´)2 and ½ Fc are indicated. (c) Illustration of IgG3 degradation by meningococcus based on identified cleavage site and time course experiment

To compare the cleavage pattern and efficacy of IgA1P toward IgG3 and IgA, a time course experiment was performed, where immunoglobulins were incubated with stationary phase growth culture supernatant of MC58 wt. Samples were analyzed by SDS-PAGE under reducing (Figure 3(a)) and non-reducing conditions (Figure 3(b)). IgA was degraded within minutes as the cleavage product was observed after 4 min when analyzed by SDS-PAGE under reducing conditions. IgG3 cleavage was considerably slower than IgA cleavage as F(ab´) and ½ Fc cleavage products first started to accumulate after 64 minutes. Under non-reducing conditions, a protein band consistent in size with single cleaved IgG3 lacking a ½ Fc-fragment gained intensity with time. This protein band is however already present in IgG3 at time point 0 min. Smaller protein bands started to appear after 64 min consistent in size with ½ Fc degradation fragments and after 128 min protein bands consistent with F(ab´)_2_ degradation fragments appeared.

Taken together with the determination of cleavage site within the IgG3 heavy chain by N-terminally sequencing, these findings are in agreement with sequential cleavage of the IgG3 heavy chains between the core and lower hinge region leading to single cleaved IgG3 (scIgG) as an intermediate and finally resulting in F(ab)2 and Fc fragments (Figure 3(c)).

### Only meningococcal strains encoding an IgA1P cleavage type 1 degrade IgG3

**Figure 4.**
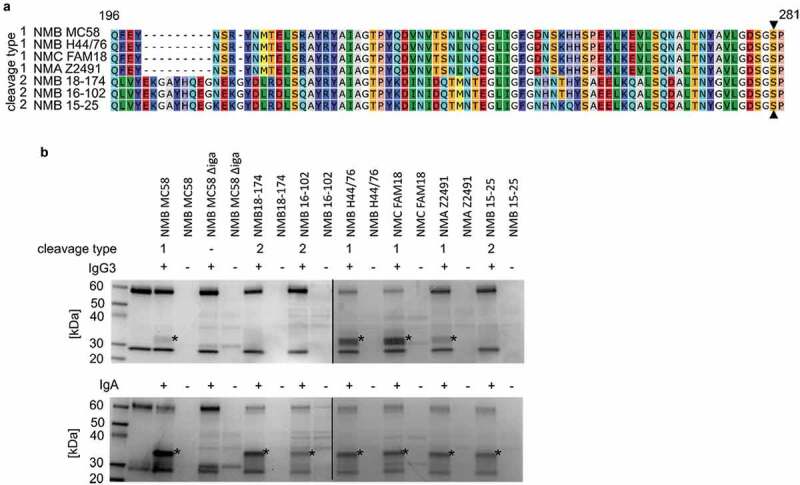
IgG3 degrading capability of different *N. meningitidis* strains. (a) Alignment of the aa 196 to 281 regions of IgA1P that defines the cleavage type of the investigated strains. The catalytic serine residue is indicated with filled triangles (▲). (b) SDS-PAGE analysis under reducing conditions of IgG3 and IgA incubated with stationary phase culture supernatants of *N. meningitidis* strains of different capsule type encoding either IgA1P cleavage type 1 or 2. Observed IgA and IgG3 degradation products are indicated with asterisks (*). The black line indicates that samples were run on separate gels in parallel

To investigate if the degradation of IgG3 is a common feature of the meningococcus, a panel of meningococcal strains of various serogroups as well as clinical isolates was selected to assess their IgG3 degradation activities. The panel consisted of strains of different serogroups; (MC58 and H44/76: serogroup B, FAM18: serogroup C and Z2491: serogroup A) and clinical isolates of serogroup B (18–174, 16–102 and 15–25), encoding for either IgA1P cleavage type 1 or IgA1P cleavage type 2. Strains were classified into IgA1P cleavage type 1 or IgA1P cleavage type 2 based on the absence or presence of the cleavage type 2 specific consensus sequence VYEKGAYHQEGNEKG in the protease domain of IgA1P within 80 aa N-terminal of the catalytic serine residue (Figure 4(a)). All tested strains encoding an IgA1P cleavage type 1 were able to degrade both IgA and IgG3, whereas all strains encoding an IgA1P cleavage type 2 were only able to degrade IgA (Figure 4(b)). Strain 18–174 (encoding IgA1P cleavage type 2) was further investigated for cleavage of all IgG subclasses and shown to only degrade IgA (Fig. S2A):

To confirm IgG3 cleavage by IgA1P cleavage type 1 is not serogroup dependent, we generated an *iga* deletion mutant in the serogroup C strain FAM18 (FAM18 *∆iga*). Supernatant of this mutant strain did not process any IgA or IgG3 degrading activity (Fig. S2B).

### Recombinant IgA1P cleavage type 1 cleaves IgG3

**Figure 5.**
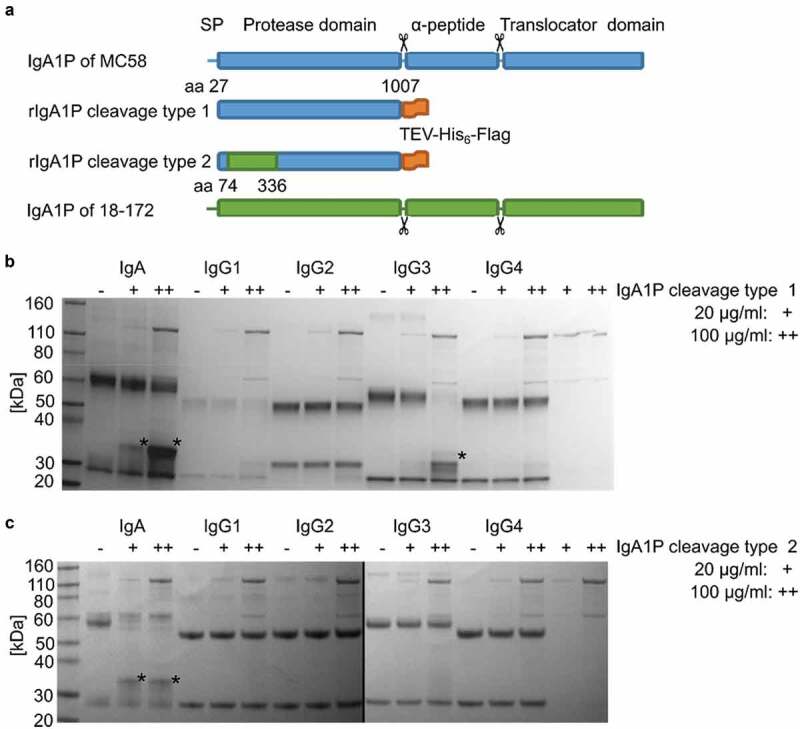
Immunoglobulin degradation assay with recombinant IgA1P (rIgA1P) cleavage type 1 and 2. (a) rIgA1P cleavage type 1 was constructed to consist of aa 27 to 1007 from IgA1P of MC58 fused to a C-terminal TEV-His_6_-Flag tag, while rIgA1P cleavage type 2, aa 74 to 336 from IgA1P of strain 18–174 exchanged the corresponding part in the previous construct. The autoproteolytic cleavage sites are indicated with scissor symbols. Reducing SDS-PAGE analysis of IgA and IgG1-4 incubated either with either 20 µg/ml (+) or 100 µg/ml (++) purified (b) rIgA1P cleavage type 1 or (c) cleavage type 2 produced in *E. coli*. Observed IgA and IgG3 degradation products are indicated with asterisks (*). The black line indicates that samples were run on separate gels in parallel

To further verify that the observed proteolytic activity of meningococci against IgG3 is directly executed by the IgA1P cleavage type 1, recombinant proteins of the protease domain of IgA1P (rIgA1P) of both cleavage type 1 and cleavage type 2 were generated. These ~111 kDa constructs were consistent with the secreted form of IgA1P generated through autoproteolytic cleavage and autotransporter mediated secretion. The rIgA1P cleavage type 1 was cloned from MC58, while rIgA1P cleavage type 2 was cloned as a chimera from the clinical isolate 18–174 and MC58 (Figure 5(a)). These C-terminal His-tagged constructs were produced in *Escherichia coli* (*E. coli*) BL21 and purified by nickel affinity chromatography (Fig. S3). Increasing concentrations of rIgA1P cleavage type 1 were incubated with IgA and IgG subclasses 1 to 4. Consistent with the previous observations in experiments with meningococcal culture supernatants (Figures 2(a) and 4(b)), degradation of IgA and IgG3 was observed (Figure 5(b)), confirming that IgG3 degradation was directly executed by IgA1P cleavage type 1. With increased concentrations of rIgA1P cleavage type 1, additional faint bands were observed indicating weak degradation of other IgG subclasses (Figure 5(b)).

To exclude that the absence of IgG3 degradation by IgA1P cleavage type 2 encoding strains was not due to general lower protease production but its cleavage specificity, rIgA1P cleavage type 2 was incubated with the immunoglobulins at the same concentrations as previously used for rIgA1P cleavage type 1 (Figure 5(a)). Again consistent with previous observations (Figure 4(b)), rIgA1P cleavage type 2 only cleaved IgA and not IgG3 (Figure 5(c)).

### IgA1P cleavage type 1 encoding meningococcal isolates are more prevalent among IMD cases than carrier cases

IgG3 antibodies have previously been shown to be highly effective in the immune defense against meningococci [29,30]. With the knowledge that meningococcal IgA1P cleavage type 1 is able to degrade IgG3, we hypothesize that the IgG3 degradation capability of meningococci encoding an IgA1P cleavage type 1 might confer an advantage during systemic infection and thereby of importance in the development of IMD. The prevalence of IgA1P cleavage type 1 and cleavage type 2 encoding isolates among IMD and carrier cases was therefore investigated.

**Figure 6.**
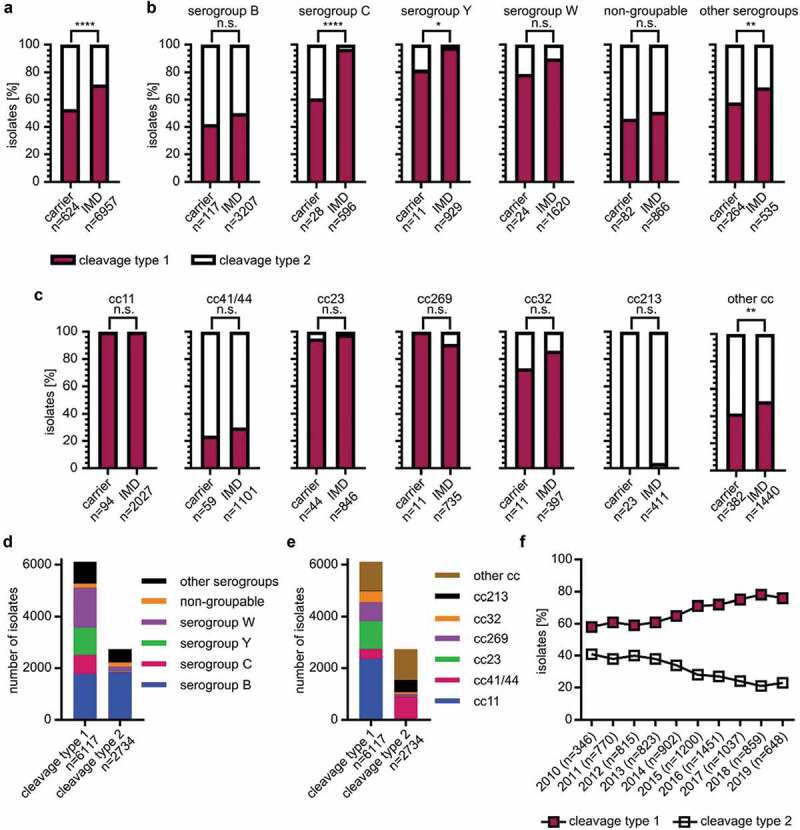
Prevalence of IgA1P cleavage type 1 and 2 encoding isolates from invasive meningococcal disease (IMD) and carrier cases collected between 2010 and 2019 in Europe. (a) Whole genome sequenced meningococcal isolates in the *Neisseria* PubMLST were based on their *iga* gene sequences classified as either IgA1P cleavage type 1 or 2 encoding isolates, separated into either from IMD and carrier case. (b) This analysis was stratified for the major serogroups (including non-groupable), as well as (c) major clonal complexes (cc). Significance differences in prevalence between isolates from IMD cases and carrier cases were assessed by Fisher’s exact test (*p* > 0.05: n.s.; *p* < 0.05: *, *p* < 0.01: **, *p* < 0.0001: ****). (d) Distribution of different serogroups and (e) cc among isolates encoding IgA1P cleavage type 1 or 2 are shown. (f) The prevalence of IgA1P cleavage type 1 encoding isolates for each year correlated to the time course as assessed by Pearson correlation (r = 0.9356; p < 0.0001). Number of isolates (*n*) for each group is given for every analysis. All numbers underlying (a-c) and (F) can be found in Table S1 and number underlying (d-e) in Table S2

Searches in the *Neisseria* PubMLST database [22] revealed a total of 8203 clinical meningococcal isolates from 2010 to 2019 in Europe with complete translated aa sequences of the *iga* gene. These aa sequences were used to classify the isolates into either IgA1P cleavage type 1 or cleavage type 2 encoding isolates. IgA1P cleavage type 1 encoding strains that are capable of degrading IgG3, are significantly (p < 0.0001) more common among isolates from IMD cases (71%; 4942 of 6957) than among isolates from carrier cases (53%, 329 of 624) (Figure 6(a) and Table S1).

Meningococci are classified into serogroups based on the composition of their capsular polysaccharides. In the serogroup stratified analysis, serogroup C (97% vs. 61%) and Y (98% vs. 82%) had significant higher prevalence (p < 0.0001 and p = 0.0253, respectively) of IgA1P cleavage type 1 encoding isolates among isolates from IMD cases than carrier cases. In serogroup B (50% vs. 42%) and W (90% vs.79%), the same trend was observed but differences were not significant (Figure 6(b), Table S1). In addition to serogroups, meningococci can also be grouped by multilocus sequence typing (MLST) into clonal complexes (cc) based on polymorphisms in seven housekeeping genes [31]. Specific cc are prevalent in outbreaks and are linked to specific serogroups. When the same analysis was performed on cc stratification, no significant differences in prevalence of IgA1P cleavage type 1 encoding isolates between IMD and carrier cases were observed (Figure 6(c), Table S1).

The investigated isolates encode more often an IgA1P cleavage type 1 (n = 6117) than IgA1P cleavage type 2 (n = 2734). Among serogroup B and non-groupable isolates, the numbers of isolates encoding for both cleavage types are similar, whereas serogroup C, Y and W isolates predominantly encode for cleavage type 1 (Figure 6(d), Table S2). When comparing the distribution of the different cc among isolates encoding IgA1P cleavage type 1 and 2, distinct different distribution patterns were observed (Figure 6(e), Table S2). For example, isolates of cc11, that are linked specifically to serogroups C and W, were shown to almost exclusively encode IgA1P cleavage type 1 (Table S2). Likewise, cc23, cc32 and cc269 were also found to encode predominately IgA1P cleavage type 1 whereas cc41/44 and cc213 encode mainly IgA1P cleaving type 2. However, among the six most prevalent cc, isolates of both cleavage types were observed. To further visualize the distribution of IgA1P cleavage types among meningococci, a phylogenetic tree of the investigated isolates was generated based on MLST profiling. This phylogenetic tree was then overlaid with metadata containing the respective IgA1P cleavage type for each isolate. The phylogenetic analysis illustrates that both cleavage types are distributed over the entire tree. Notably, there is clear association of specific cc such as cc11 and cc23 to cleavage type 1, and cc213 to cleavage type 2 (Fig. S4).

Interestingly, the overall prevalence of IgA1P cleavage type 1 encoding isolates has increased significantly compared to cleavage type 2 encoding isolates during the last decade from 58% to 76% (Figure 6(f), Table S1).

## Discussion

In this study, we describe for the first time that the meningococcal IgA1-specific serine protease degrades human IgG3 in addition to IgA1. The degradation of IgA1 by IgA1P reflects well the adaptation of the meningococcus to its habitual niche in the human nasopharynx. The meningococcus is equipped to thwart the innate and naïve immune system, as it produces a polysaccharide capsule and factor H binding protein, in addition to IgA1P [4]. However, during invasive infections, meningococci may also encounter the adaptive immune system, including specific antibodies of the IgG3 subclass. Despite the low abundance of IgG3 (7%) compared to other IgG subclasses in serum, recent studies emphasize that IgG3 plays a critical role in the immune defense against several pathogens [30], notably meningococci [5]. Selective subnormal IgG3 levels are associated with increased susceptibility for severe and recurrent bacterial respiratory tract infections [32], as well as mortality in patients admitted to intensive care units due to pneumonia [33].

Specific antibodies of the IgG3 subclass have been shown to be the most effective initiators of the classical complement pathway on the meningococcal cell surface *in vitro*, particularly when targeting sparsely distributed antigens [29]. Moreover, IgG3 is usually one of the first immunoglobulins produced upon infection and encounter of a protein antigen [34]. The low levels, but high potency, as well as the early production during an immune response, make IgG3 an important proteolytic target of meningococci for immune evasion. The discovered cleavage of IgG3 by the meningococcal IgA1P between the core and lower hinge region can potentially abrogate the major IgG3-mediated immune responses, as the antigen-binding part of the antibody is separated from the effector-binding Fc region. Despite the slower cleavage rate of IgA1P toward IgG3 than IgA1 (Figure 3), IgG3 cleavage might still be sufficient to contribute to immune evasion as IgG3 concentrations are lower than IgA1 in both serum (39 µg/ml vs 71 µg/ml) [35] and mucosal secretions such as saliva (15 µg/ml vs 72 µg/ml) [36]. Furthermore, it has been shown that cleavage of only one heavy chain in the hinge region per IgG molecule is sufficient to render it inactive [37].

The presence of IgA1P-specific antibodies in pooled human serum highlights that IgA1P is produced by the meningococcus during colonization or infection of the human host (Fig. S6). These antibodies may hamper or neutralize the enzymatic function of IgA1P. In individuals susceptible to meningococcal infections, these antibodies are most likely not present. However, during colonization and infection, anti-meningococcal antibodies including IgA1P-neutralizing antibodies might develop. According to the current dogma of infection, pathogen specific IgG3 will develop first prior to other pathogen specific IgG subtypes [34] and thereby would explain the advantage of strains expressing IgA1P cleavage type 1. However, further investigation is warranted as the involvement of IgA1P-mediated IgG3 cleavage in immune evasion is not fully demonstrated at this stage.

Due to an exon quadruplication, IgG3 consists of an elongated hinge region of 62 aa, compared to 12 aa or 15 aa of other IgG subclasses [38]. This elongated hinge makes IgG3 putatively prone to proteolysis. Yet, the short half-life of IgG3 can be attributed to a single aa difference in the Fc region of the heavy chain and not to the proteolytic vulnerability of IgG3 [39]. The unique hinge region of IgG3 consists of 21 proline and 11 cysteine residues that form the disulfide bonds between the two heavy chains [40]. All described substrates of IgA1P are proline-rich, the novel cleavage motif of the meningococcal IgA1P within the IgG3 hinge (CP*AP) (Figure 2(c)) does not match the so far described cleavage motifs in other substrates (PP*(T/S/A)P). Instead of a proline, a cysteine residue is found in the P_2_ position of the cleavage motif in IgG3. This motif (CPAP) is also found in the hinge region of other human IgG subclasses and may be cleaved by IgA1P as exemplified in Figure 5(b). However, residues outside the cleavage motif or the accessibility of the cleavage motif might also contribute to susceptibility of substrates to proteolytic cleavage by IgA1P. Indeed, it has been shown in case of IgA1 cleavage by different IgA1 proteases, that cleavage specificity is not mediated by the cleavage motif itself but by the hinge length and structures in the Fc-region [41,42].

Previously, the only known functional difference between the meningococcal IgA1P cleavage type 1 and cleavage type 2 was the different cleavage sites within the IgA1 hinge two aa apart from each other. Here we show that the IgA1P cleavage type 1 can additionally cleaves IgG3, while the cleavage type 2 does not. Besides a few single aa polymorphisms, IgA1P cleavage type 2 has in comparison to IgA1P cleavage type 1 an additional ten aa in the region 80 aa toward the N-terminal from the catalytic serine residue. These additional aa might lead to a slight structural change of the active site cleft affecting the cleavage specificity. Indeed, our *in silico* predicted overlay structure of meningococcal IgA1P cleavage type 1 and 2 based on a crystal structure of *Haemophilus influenzae* IgA1 specific serine protease indicates that these additional aa present in IgA1P cleavage type 2 might locate close to the active site cleft (Fig. S5). Moreover, all tested meningococcal strains encoding an IgA1P cleavage type 1 were able to degrade IgG3, while IgA1P cleavage type 2 encoding strains were not able to do so (Figure 4).

The biological importance of this IgG3 degradation by the meningococcus is difficult to investigate as there is a lack of suitable infection models to study meningococcal evasion from the human humoral immunity. Also *in vitro* assays, such as serum survival assays, are difficult to adopt, as adequate amounts of pathogen-specific IgG3 in relation to other antibodies have to be present. Presence of IgA1P-specific and neutralizing antibodies can further complicate such analysis. Therefore, a molecular epidemiological approach similar to Karlsson *et al*. [43], taking advantage of the different IgG3 degrading abilities of IgA1P cleavage type 1 and 2 encoding isolates was applied in this study. Meningococcal IgA1P cleavage type 1 production has been suggested to correlate with epidemic pathogenicity [44]. In this study, an overall significant higher prevalence of IgA1P cleavage type 1 encoding isolates among isolates from IMD cases compared to isolates from carriers was observed (Figure 6(a), Table S1). This suggests that IgA1P cleavage type 1 encoding isolates could indeed possess higher degree of pathogenicity due to their IgG3–degrading ability. Similar observations (*i.e*. cleavage type 1 more prevalent among IMD) were also apparent when the isolates were stratified into serogroups (Figure 6(b)). However, differences cannot be observed within individual cc (Figure 6(c)). As IgA1P cleavage types are niched into specific cc, low number of isolates of the respective minor cleavage types hindered statistical evaluation. Also major cc are often associated to outbreaks; the corresponding low number of isolates from carrier cases might be a reason why no significant differences within individual cc were observed.

Even though certain cc preferentially encode distinct cleavage types, both cleavage types were found in most clades in our phylogenetic analysis of the investigated isolates (Fig. S4). This indicates that cleavage types are not fixed within different meningococcal lineages. Interestingly, cc11 is almost exclusively harboring IgA1P cleavage type 1. Meningococci of serogroup W cc11 has become an increasingly successful clade causing IMD on a global scale [45,46], tracing back to an outbreak related to the Hajj in 2000 [47]. While our results cannot confirm a causative relationship, serogroup W cc11 propensity for causing atypical, but highly severe IMD [48] and the almost exclusive maintenance of IgA1P cleavage type 1 remains an interesting observation.

Moreover, our study here shows an increasing prevalence of isolates encoding IgA1P cleavage type 1 compared to cleavage type 2 during the observed decade (2010–2019) in Europe (Figure 6(f)). This might be indicative of an evolutionary advantage given by the IgG3 degrading capability. During the investigated time period (2010–2019), the meningococcal B (4CMenB) vaccine Bexsero was introduced in several European countries [49]. The corresponding decrease in prevalence of serogroup B isolates might conversely increase the prevalence of IgA1P cleavage type 1 encoding isolates as serogroup B is associated to a higher degree with IgA1P cleavage type 2 than cleavage type 1 (Figure 6(d)). The overall increased association of IgA1P cleavage type 1 encoding isolates with IMD could also be caused by other undescribed functional differences. IgA1P cleavage type 1 in combination with other virulence factors such as polysaccharide capsule and factor H binding protein could potentially play a role with the increased association to IMD.

In conclusion, our molecular epidemiological findings here showing higher prevalence of IgG3 degrading IgA1P cleavage type 1 encoding isolates among IMD cases compared to carrier cases underscores the biological importance of the described degradation of IgG3 by meningococci.

## Supplementary Material

Supplemental Material
