# Supplementary material for: Neisseria meningitidis IgA1-specific serine protease exhibits novel cleavage activity against IgG3
Source: Virulence. 2021 Jan 18;12(1):389–403. doi: 10.1080/21505594.2021.1871822 (PMC7834093; doi:10.1080/21505594.2021.1871822)
Supplement: Supplemental Material [file KVIR_A_1871822_SM6012.docx]

## **Supplemental data**


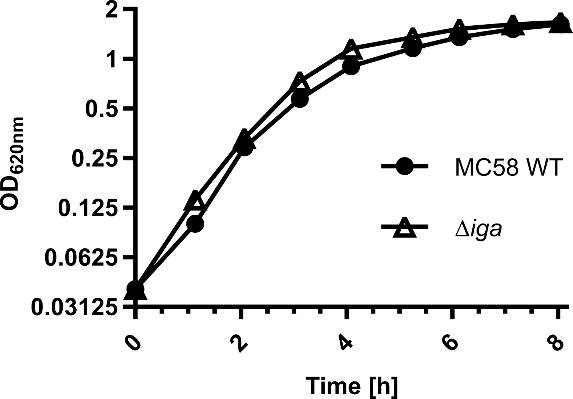


**Fig. S1. Growth curves of MC58 wt (solid circles) and a *∆iga* mutant (open triangle) in BHI.**


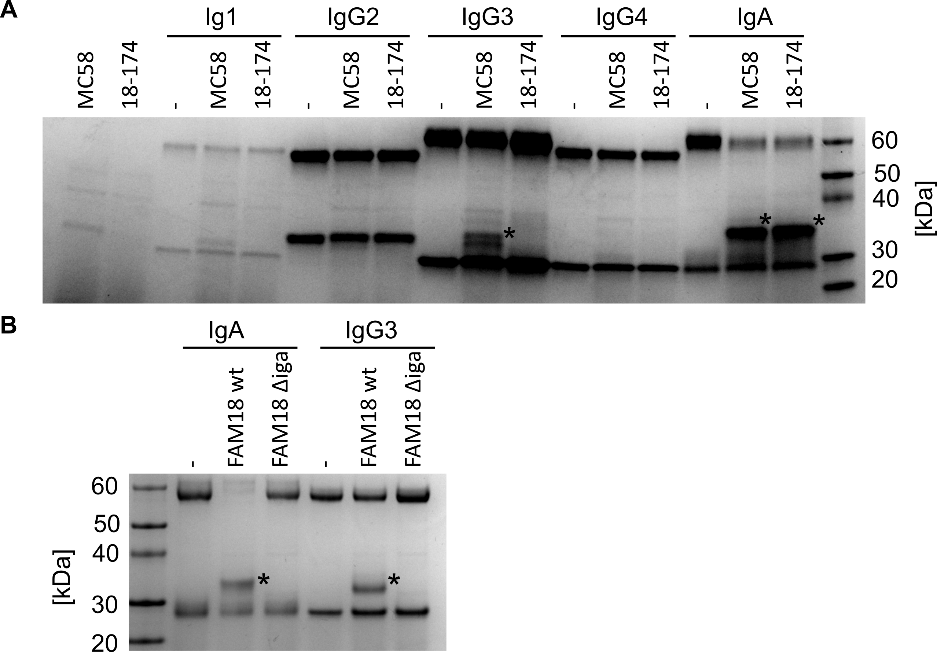


**Fig. S2. Immunoglobulin degradation assays with meningococcal culture supernatants.**

(A) SDS-PAGE analysis under reducing conditions of IgG1-4 and IgA incubated with stationary phase culture supernatants of *N. meningitidis* MC58 (encoding IgA1P cleavage type 1) and 18-174 (encoding IgA1P cleavage type 2). (B) SDS-PAGE analysis under reducing conditions of IgG3 and IgA incubated with stationary phase culture supernatants of *N. meningitidis* FAM18 and FAM18 *∆iga*. Observed IgA and IgG3 degradation products are indicated with asterisks (*).


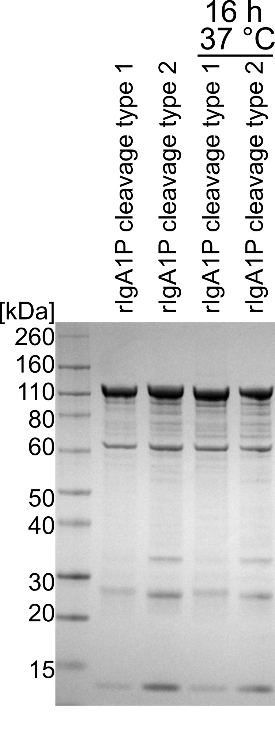


**Fig. S3. SDS-PAGE analysis of purified rIgA1P (5 µg) cleavage type 1 and 2 prior and post incubation at 37°C for 16 hours.**

The ̴110 kDa protein bands represent the full length recombinant proteins, while other protein bands represent co-purified impurities.


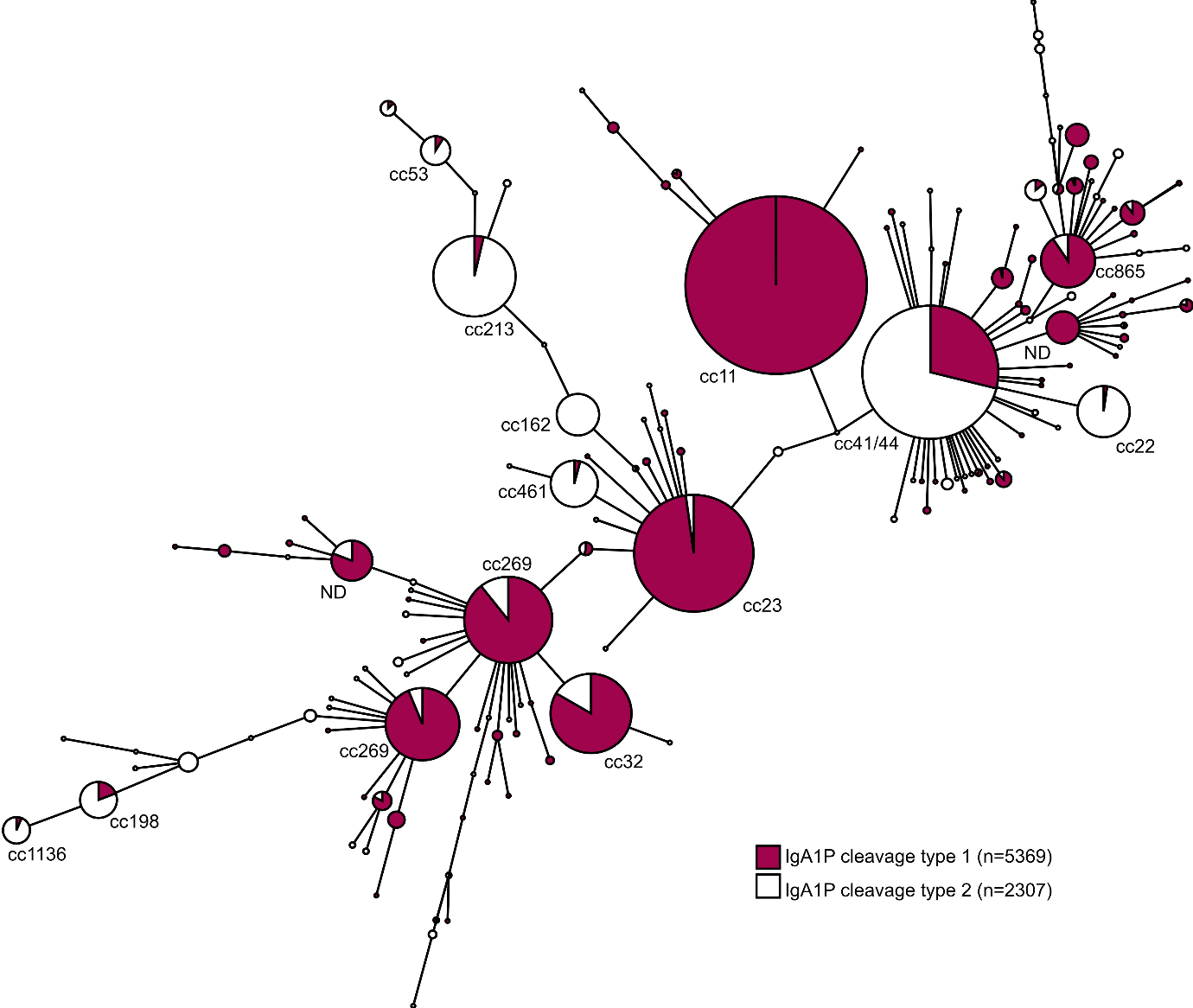


**Fig. S4. Phylogenetic tree of meningococcal isolates based on MLST profiles and their corresponding IgA1P cleavage types.**

Unrooted minimum spanning tree based on MLST profiles showing the phylogenetic relationship between isolates from both carrier and IMD cases in Europe during the years 2010 to 2019. Node sizes correspond to number of isolates in the same node. All nodes with more than 50 isolates are annotated with their corresponding cc and incomplete data denoted as ND. Prevalence of IgA1P cleavage types within nodes is illustrated by pie charts (violet for cleavage type 1 and white for cleavage type 2).


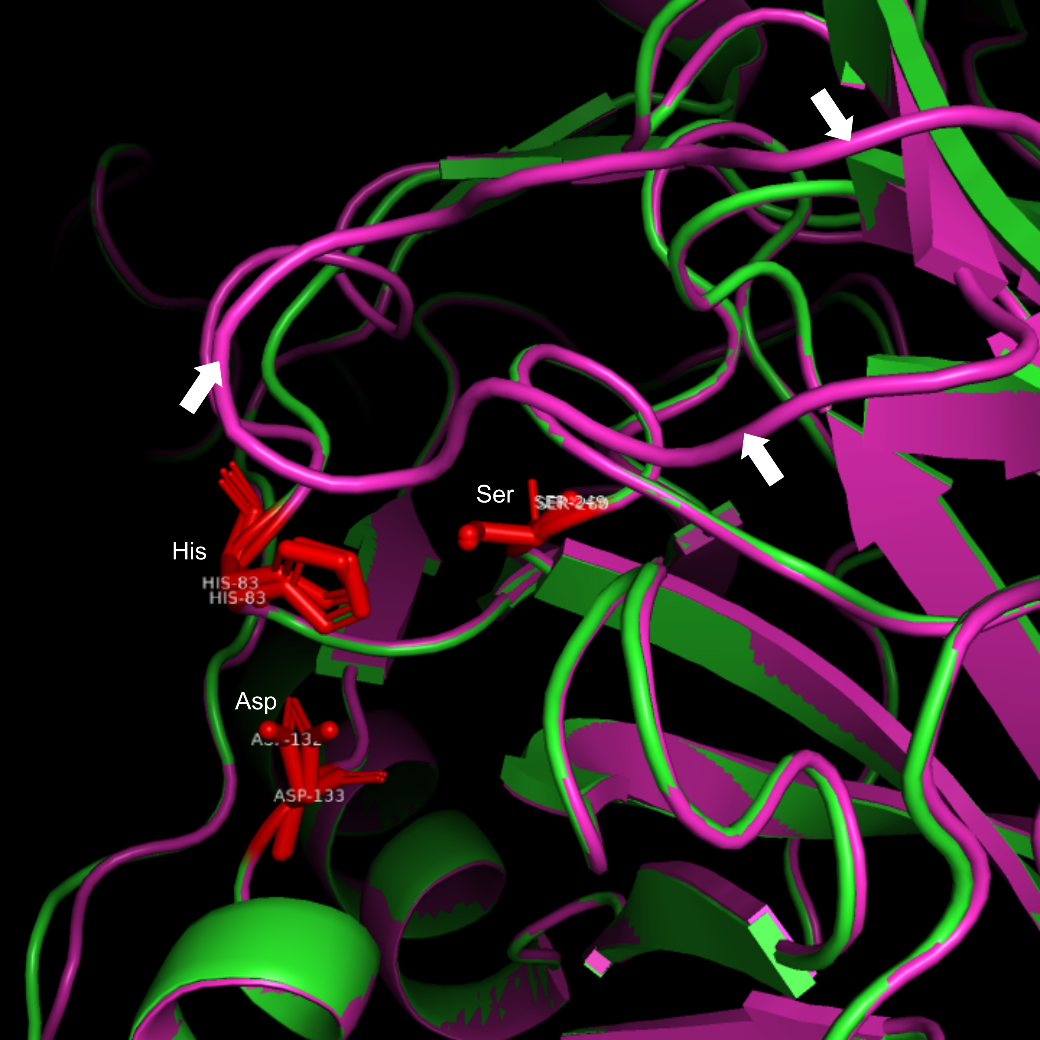


**Fig. S5. *In silico* modelled overlay structure of meningococcal IgA1P cleavage type 1 and 2.**

IgA1P cleavage type 1 (green) and 2 (violet) were *in silico* modelled by SWISS-MODEL based on a crystal structure of *Haemophilus influenzae* IgA1 specific serine protease. The selection shows the active site cleft with the catalytic site residues histidine (His), aspartic acid (Asp) and serine (Ser) in red and the additional ten aa only found in IgA1P cleavage type 2 (indicated with white arrows).

**Fig. S6. Western blot analysis of meningococcal cell lysates and recombinant IgA1P using pooled human serum as primary antibody.**


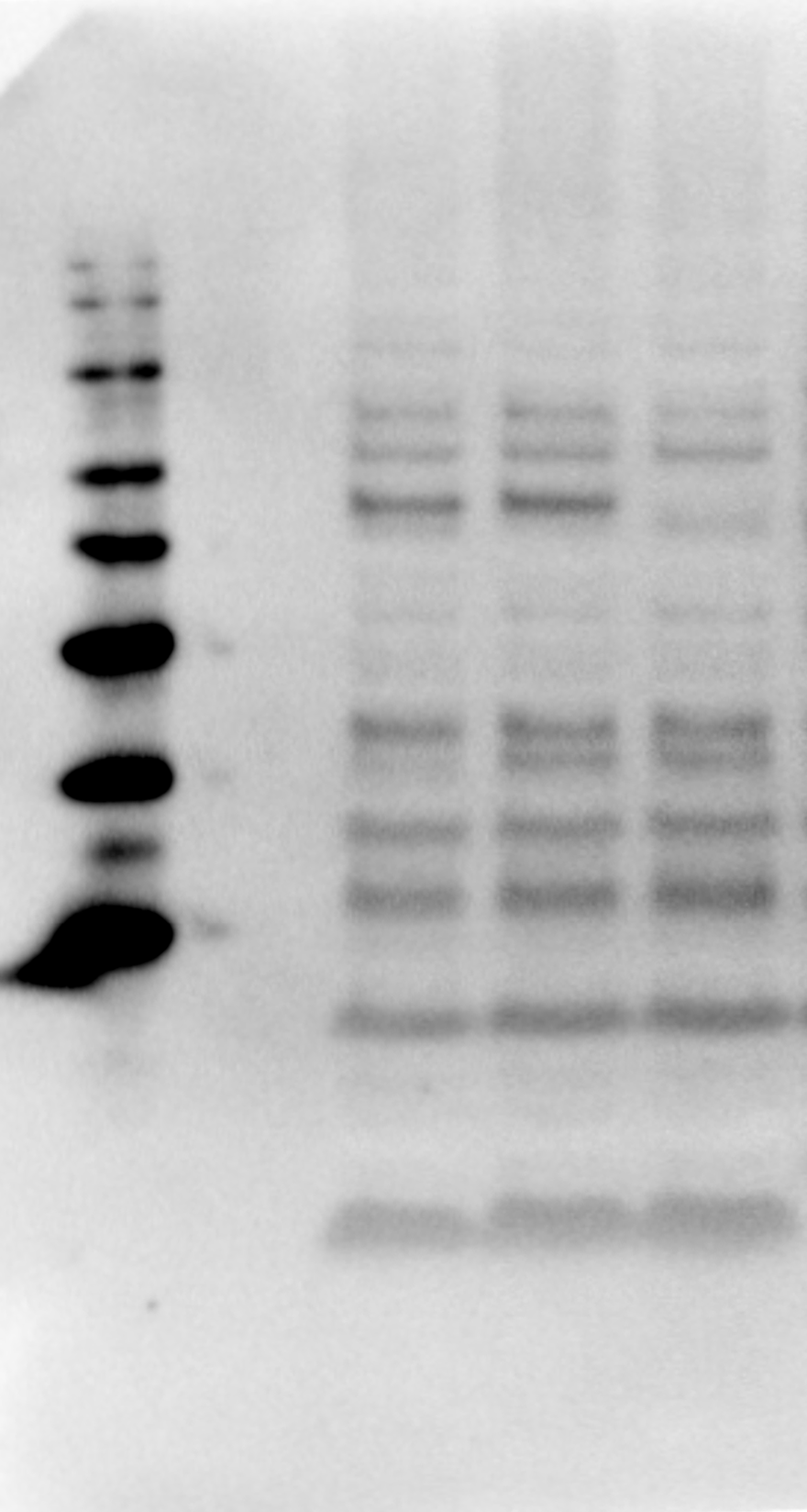


[**kDa**]

120

100

60

50

40

30

20

*****


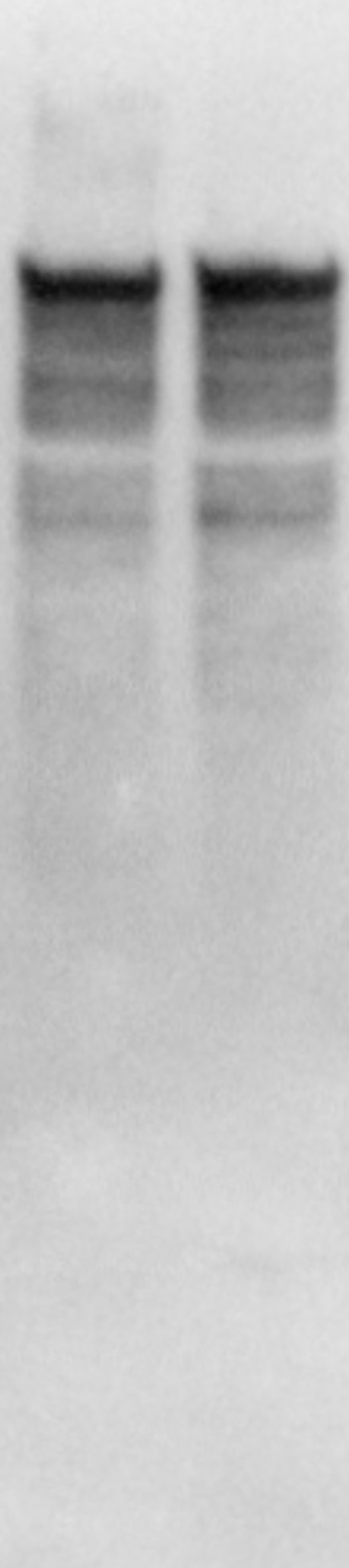


**MC58 wt**

**MC58 *∆cssD***

**MC58 *∆cssD∆iga***

**rIgA1P cleavage type 1**

**rIgA1P cleavage type 2**

80

A major immunoreactive band (*) is absent in the cell lysate of MC58 *ΔcssDΔiga* mutant compared to cell lysate of wt and *ΔcssD* mutant. Both rIgA1P cleavage type 1 and 2 are detected with the pooled human serum.

**Table S1**

|  | | **Number of isolates** | | **Percentage of isolates (within same row)** | | **Fisher's exact test**  **(two-sided)** |
| --- | --- | --- | --- | --- | --- | --- |
|  |  | cleavage type 1 | cleavage type 2 | cleavage type 1 | cleavage type 2 | *P*-value |
| All | carrier | 329 | 295 | 53% | 47% | <0.0001 |
|  | IMD | 4942 | 2015 | 71% | 29% |  |
| Serogroup B | carrier | 49 | 68 | 42% | 58% | 0.1094 |
|  | IMD | 1594 | 1613 | 50% | 50% |  |
| Serogroup C | carrier | 17 | 11 | 61% | 39% | <0.0001 |
|  | IMD | 580 | 16 | 97% | 3% |  |
| Serogroup Y | carrier | 9 | 2 | 82% | 18% | 0.0253 |
|  | IMD | 909 | 20 | 98% | 2% |  |
| Serogroup W | carrier | 19 | 5 | 79% | 21% | 0.0955 |
|  | IMD | 1454 | 166 | 90% | 10% |  |
| non-groupable | carrier | 83 | 97 | 46% | 54% | 0.4826 |
|  | IMD | 36 | 34 | 51% | 49% |  |
| other serogroups | carrier | 152 | 112 | 58% | 42% | 0.0016 |
|  | IMD | 369 | 166 | 69% | 31% |  |
| cc11 | carrier | 94 | 0 | 100% | 0% | >0.9999 |
|  | IMD | 2026 | 1 | 100% | 0% |  |
| cc41/44 | carrier | 14 | 45 | 24% | 76% | 0.3801 |
|  | IMD | 329 | 772 | 30% | 70% |  |
| cc23 | carrier | 42 | 2 | 95% | 5% | 0.2408 |
|  | IMD | 829 | 17 | 98% | 2% |  |
| cc269 | carrier | 11 | 0 | 100% | 0% | 0.6121 |
|  | IMD | 671 | 64 | 91% | 9% |  |
| cc32 | carrier | 8 | 3 | 73% | 27% | 0.1884 |
|  | IMD | 343 | 54 | 86% | 14% |  |
| cc213 | carrier | 0 | 23 | 0% | 100% | >0.9999 |
|  | IMD | 16 | 395 | 4% | 96% |  |
| other cc | carrier | 160 | 222 | 42% | 58% | 0.0027 |
|  | IMD | 728 | 712 | 51% | 49% |  |
| Year of isolation  (year of investigation) | 2010 (1 y) | 202 | 144 | 58% | 42% |  |
|  | 2011 (2 y) | 471 | 299 | 61% | 39% |  |
|  | 2012 (3 y) | 486 | 329 | 60% | 40% |  |
|  | 2013 (4 y) | 508 | 315 | 62% | 38% |  |
|  | 2014 (5 y) | 589 | 313 | 65% | 35% |  |
|  | 2015 (6 y) | 855 | 345 | 71% | 29% |  |
|  | 2016 (7 y) | 1054 | 397 | 73% | 27% |  |
|  | 2017 (8 y) | 782 | 255 | 75% | 25% |  |
|  | 2018 (9 y) | 676 | 183 | 79% | 21% |  |
|  | 2019 (10 y) | 494 | 154 | 76% | 24% |  |
| Pearson correlation | R (95% confidence interval) |  | | 0.9632  (0.8476 to 0.9915) | |  |
|  | *P*-value  (two-tailed) |  |  | <0.0001 | |  |

**Table S1. Number and percentage of IgA1P cleavage type 1 and cleavage type 2 encoding strains among isolates from carriers and IMD cases.**

**Table S2**

|  | | **Serogroups** | | | | | | | | |
| --- | --- | --- | --- | --- | --- | --- | --- | --- | --- | --- |
|  |  | B | C | Y | W | non-groupable | | other serogroups | |  |
| Number of isolates | cleavage type 1 | 1767 | 751 | 1052 | 1544 | 142 | | 861 | |  |
|  | cleavage type 2 | 1819 | 30 | 24 | 186 | 148 | | 527 | |  |
| Percentage of isolates (within same row) | cleavage type 1 | 28.9% | 12.3% | 17.2% | 25.2% | 2.3% | | 14.1% | |  |
|  | cleavage type 2 | 66.5% | 1.1% | 0.9% | 6.8% | 5.4% | | 19.3% | |  |
|  | | | | | | | | | | |
|  | | **Clonal complexes** | | | | | | | | |
|  |  | cc11 | cc41/  44 | cc23 | cc269 | cc32 | cc213 | | other cc | |
| Number of isolates | cleavage type 1 | 2361 | 384 | 1074 | 738 | 426 | 16 | | 1118 | |
|  | cleavage type 2 | 2 | 898 | 22 | 66 | 66 | 486 | | 1194 | |
| Percentage of isolates (within same row) | cleavage type 1 | 38.6% | 6.3% | 17.6% | 12.1% | 7.0% | 0.3% | | 18.3% | |
|  | cleavage type 2 | 0.1% | 32.8% | 0.8% | 2.4% | 2.4% | 17.8% | | 43.7% | |

**Table S2. Number and percentage of isolates with different serogroups and clonal complexes among IgA1P cleavage type 1 and cleavage type 2 encoding strains.**

**Sequences used for *in silico* modeling of IgA1P**

>IgA1P Protease domain cleavage type 1 from FAM18, H83 D132 S249

YALTPYSEAALVRDDVDYQIFRDFAENKGKFFVGATDLSVKNKQGQNIGNALSNVPMIDFSVADVNRRTLTVIDPQYAVSVK**H**VKGDEISYYGHHNGHLDVSNDENEYRSVAQNDYEPNKNWHHGNQGRLE**D**YNMARLNKFVTEVAPIAPTSAGGGVETYKDKNRFSEFVRVGAGTQFEYNSRYNMTELSRAYRYAIAGTPYQDVNVTSNLNQEGLIGFGDNSKHHSPEKLKEVLSQNALTNYAVLGD**S**GSPLFAYDKQEKRWVFLGAYDYWAGYQKNSWQEWNIYKKEFADKIKQRDNAGTIKGNGEHHWNITFGTNSKIGSTAVRLAGNEKDANNGQNVTFEDNGTLVLDQNINQGAGGLFFKGDYTVKGINNDITWLGAGIDVTDGKKVVWQVKNPNGDRLAKIGKGTLEINGTGVNQGQLKVGDGTVILNQQADADKKVQAFSQVGIVSGRGTLVLNSSNQINPDNLYFGFRGGRLDANGNDLTFEHIRNVDEGARIVNHNTSHASTITLTGKSLITNPNSLSVHSIQNDYDEDDYSYYYRPRRPIPQGKDLYYKNYRYYALKSGGSVNAPMPENGVTENNDWVFMGYTQEEAKKNAMNHKNNQRISGFSGFFGEENGKGHNGALNLNFNGKSAQNRFLLTGGTNLNGKISVTQGNVLLSGRPTPHARDFVNKSSARKDAHFSKNNEVVFEDDWINRTFKAAEIAVNQSASFSSGRNVSNITANITATDNAKVNLGYKNGDEVCVRSDYTGYVTCNTGNLSDKALNSFGATQINGNVNLNQNAALVLGKAALWGQIQGQGNSRVSLNQHSKWHLTGD

>IgA1P Protease domain cleavage type 2 from FAM18/18-174-chimera, H83 D132 S260

YALTPYSEAALVRDDVDYQIFRDFAENKGKFFVGATDLSVKNKQGQNIGNALSNVPMIDFSVADVNKRIATVVDPQYAVSVK**H**AKAEVHTFYYGQYNGHNDVADKENEYRVVEQNNYEPHKAWGASNLGRLE**D**YNMARFNKFVTEVAPIAPTDAGGGLDTYKDKNRFSSFVRVGAGRQLVYEKGAYHQEGNEKGYDLRDLSQAYRYAIAGTPYKDINIDQTMNTEGLIGFGNHNTHYSAEELKQALSQDALTNYGVLGD**S**GSPLFAFDKQKNQWVFLGTYDYWAGYGKKSWQEWNIYKKEFADEIKQRDNAGTIKGNGEHHWNITFGTNSKIGSTAVRLAGNEKDANNGQNVTFEDNGTLVLDQNINQGAGGLFFKGDYTVKGINNDITWLGAGIDVTDGKKVVWQVKNPNGDRLAKIGKGTLEINGTGVNQGQLKVGDGTVILNQQADADKKVQAFSQVGIVSGRGTLVLNSSNQINPDNLYFGFRGGRLDANGNDLTFEHIRNVDEGARIVNHNTSHASTITLTGKSLITNPNSLSVHSIQNDYDEDDYSYYYRPRRPIPQGKDLYYKNYRYYALKSGGSVNAPMPENGVTENNDWVFMGYTQEEAKKNAMNHKNNQRISGFSGFFGEENGKGHNGALNLNFNGKSAQNRFLLTGGTNLNGKISVTQGNVLLSGRPTPHARDFVNKSSARKDAHFSKNNEVVFEDDWINRTFKAAEIAVNQSASFSSGRNVSNITANITATDNAKVNLGYKNGDEVCVRSDYTG
